# Three-Dimensional Printing of Poly-L-Lactic Acid Composite Scaffolds with Enhanced Bioactivity and Controllable Zn Ion Release Capability by Coupling with Carbon-ZnO

**DOI:** 10.3390/bioengineering10030307

**Published:** 2023-02-28

**Authors:** Xun Yuan, Wei Zhu, Zhongyuan Yang, Feng Chen, Xiaoxiao Han

**Affiliations:** National Engineering Research Center for High-Efficiency Grinding, College of Mechanical and Vehicle Engineering, Hunan University, Changsha 410082, China

**Keywords:** bioactivity, C–ZnO, 3D printing, poly-L-lactic acid, bone regeneration

## Abstract

Poly-L-lactic acid (PLLA) has gained great popularity with researchers in regenerative medicine owing to its superior biocompatibility and biodegradability, although its inadequate bioactivity inhibits the further use of PLLA in the field of bone regeneration. Zinc oxide (ZnO) has been utilized to improve the biological performance of biopolymers because of its renowned osteogenic activity. However, ZnO nanoparticles tend to agglomerate in the polymer matrix due to high surface energy, which would lead to the burst release of the Zn ion and, thus, cytotoxicity. In this study, to address this problem, carbon–ZnO (C–ZnO) was first synthesized through the carbonization of ZIF-8. Then, C–ZnO was introduced to PLLA powder before it was manufactured as scaffolds (PLLA/C–ZnO) by a selective laser sintering 3D printing technique. The results showed that the PLLA/C–ZnO scaffold was able to continuously release Zn ions in a reasonable range, which can be attributed to the interaction of Zn–N bonding and the shielding action of the PLLA scaffold. The controlled release of Zn ions from the scaffold further facilitated cell adhesion and proliferation and improved the osteogenic differentiation ability at the same time. In addition, C–ZnO endowed the scaffold with favorable photodynamic antibacterial ability, which was manifested by an efficient antibacterial rate of over 95%.

## 1. Introduction

Repairing massive segmental bone defects caused by accidents, infections, or the removal of tumors is currently one of the most pressing biomedical issues globally [1,2]. Fortunately, bone regeneration and healing have entered a new era, thanks to the progress of tissue engineering [3,4]. As a fundamental part of tissue engineering, scaffolds provide cells a place to attach and help control the behavior of cells [5,6,7]. Due to its biocompatibility and inherent biodegradability, poly-L-lactic acid (PLLA) is among the most promising of the proposed scaffold materials [8,9,10]. It can be broken down into lactic acid in the human body, which is then entirely metabolized into water and CO_2_ to be excreted in the urine and breath [11]. In addition, PLLA has been approved for biomedical uses, including tissue-engineering scaffolds, by the Food and Drug Administration [11,12]. However, the inadequate bioactivity of PLLA hinders its use in bone repair.

Zn is essential for several biochemical functions, including cell division, cell development, wound healing, and stimulating new bone formation by limiting osteoclast growth and increasing osteoblast proliferation [13,14]. Due to its capability of enhancing the development of osteoblasts, Zn has received increasing research attention for bone regeneration. In recent years, pure Zn and Zn alloys have been developed as bone implant materials, which have exhibited a favorable osteogenic effect [15,16]. Qin et al. fabricated porous Zn-Mg alloy scaffolds using laser powder bed fusion technology. The results indicated that the osteogenic ability of rabbit femurs was improved after a 6-week and 12-week implantation of a Zn-Mg scaffold [17]. Zhong et al. co-assembled Zn and Sr into HA using a collagen template biomimetic technique. The results indicated that Zn could directly stimulate the repair of bone defects at an early stage [18]. According to the available data, Zn appears to enhance bone production and regeneration, but only within a narrow dose range, so that too little or too much Zn is useless [19]. Zn has also been used as an osteogenic active agent in biomaterials, which holds tremendous promise for enhancing their bioactivity [20]. Zinc oxide (ZnO), as an important Zn source, possesses good biocompatibility and has been approved as a biomedical material by the Food and Drug Administration [21,22]. Moreover, ZnO can generate reactive oxygen species (ROS) under ultraviolet and visible light to destroy the cell membranes of bacteria [23,24]. However, ZnO nanoparticles readily aggregate in the polymer matrix because of the high surface energy, which would lead to the burst release of Zn ions, thereby inducing cytotoxicity [25].

The calcination of metal-organic frameworks (MOF) has proved an efficient way to anchor metal oxides in a carbon matrix with improved dispersion uniformity and stability [26,27]. Chen et al. successfully prepared Mn_2_O_3_ embedded into a carbon matrix with high dispersion, using MOF as sacrificial templates [28]. Guo et al. used Ce–MOF as a dispersion precursor to uniformly encapsulate CeO_2_ in a carbon matrix [29]. Zeolitic imidazolate framework-8 (ZIF-8) is formed in zeolite topologies with Zn ions and imidazolate ligands, which can be calcined to form nanostructures consisting of N-doped carbon matrices and uniformly dispersed ZnO nanoparticles [30]. The derived nanomaterials have been used in adsorption, catalytic degradation, and drug delivery [31]; however, applications for the controllable release of Zn ions in bone implant material have not been reported.

As reported, the N–doped carbon matrix may construct a chemical bond with ZnO during calcination, ensuring the continued release of Zn ions [32]. For example, Kang et al. used a metal-organic framework of MIL-88-Fe as a self-template to construct N-doped Fe_7_S_8_ embedded in N-doped carbon and confirmed that Fe-N and C-N bonds were formed during the calcination process [33]. Moreover, it is hypothesized that carbon nanostructures can offer firm binding sites to cell membrane receptors, as well as the substantial adsorption and storage of nutrients [34,35]. Interestingly, a carbon matrix as an electron acceptor could capture the photogenerated electrons in ZnO, thereby enhancing its photodynamic antibacterial ability [36,37,38].

In this work, to address the issue of insufficient bioactivity of the PLLA scaffold, carbon–ZnO (C–ZnO) nanoparticles were constructed via the calcination of ZIF-8, and ZnO was anchored to the carbon sheet. Then, C–ZnO was mixed with PLLA powder before it was manufactured into PLLA-based (PLLA/C–ZnO) composite scaffolds via selective laser sintering (SLS) using three-dimensional (3D) printing technology. The morphology, phase structure, and chemical composition of C–ZnO were assessed. The Zn ion release behavior from the PLLA/C–ZnO scaffolds was thoroughly investigated. In addition, the influence of the controllable Zn ions on cell adhesion, proliferation, and osteogenic capacities was uncovered. The photodynamic antibacterial capacity was also tested.

## 2. Materials and Methods

### 2.1. Materials

Zinc nitrate hexahydrate (Zn(NO_3_)_2_·6H_2_O), 2-methylimidazole (2-MIM), and methanol (CH_3_OH) were obtained from Sigma Chemical Co. (Shanghai, China). PLLA powder was purchased from Shenzhen Polymtek Biomaterial Co. Ltd. (Shenzhen, China).

### 2.2. C–ZnO Preparation

**Figure 1:**
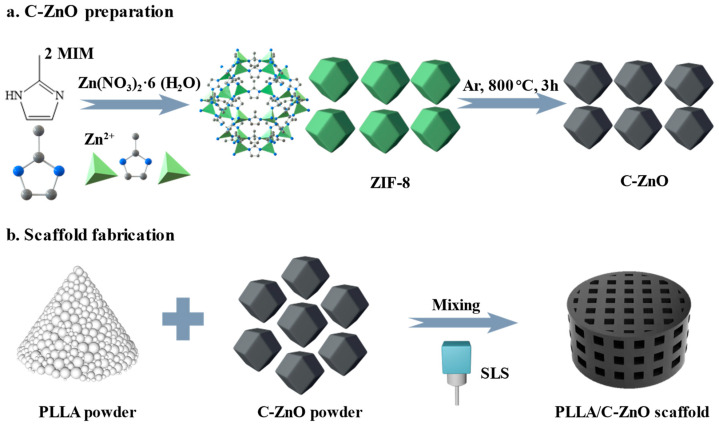
Schematic illustration.

A schematic illustration of ZIF-8 and C–ZnO preparation is shown in Figure 1a. Initially, 2.8 g of Zn(NO_3_)_2_·6H_2_O powder and 3.1 g of 2-MIM powder were separately dispersed in 50 mL methanol and stirred for 5 min. After complete dissolution, the 2-MIM solution was rapidly added into Zn(NO_3_)_2_·6H_2_O solution under intense agitation. Subsequently, the mixed solution was sealed and placed in an oven (35 °C) for 2 h. Finally, the ZIF-8 precipitate was cleaned 3 times with methanol and dried at 80 °C for 12 h. C–ZnO powder was prepared using the direct carbonization method. Briefly, 0.5 g ZIF-8 powder was subjected to a tube furnace at 800 °C, with a heating rate of 5 °C min^−1^, for 3 h under an Ar flow. Finally, the black C–ZnO powder was obtained without further processing.

### 2.3. Scaffold Fabrication

The schematic diagram of the scaffold fabrication is shown in Figure 1b. Initially, 0.2 g C–ZnO powder and 9.8 g PLLA powder (2 wt % ZnO) were separately dispersed in 30 mL of anhydrous ethanol. Then, the ZnO solution was poured into the PLLA solution, followed by sonication and stirring for 20 min. Afterward, the mixed suspension was dried and ground to yield the PLLA/C–ZnO powder. Finally, the PLLA/C–ZnO scaffold was fabricated using a homemade SLS machine, which is equipped with a CO_2_ laser with a beam spot size of ~200 µm. SLS is a new additive manufacturing technology. Its advantage is that it can achieve the personalized manufacturing of three-dimensional porous bone scaffolds, as required. During processing, the mixed powder is first preheated (to slightly lower than the powder’s melting point). Afterward, the powder is selectively sintered to create a layer based on a predesigned model. Then, a layer of new powder is laid down, after which sintering continues to pile the material up layer by layer to process it into a bone scaffold. In the current study, the laser intensity, scan speed, and layer thickness were set at 6.5 W, 220 mm/s, and 150 µm, respectively, based on the parameter optimization results. The as-obtained product was denoted as the PLLA/C–ZnO scaffold. In the meantime, pure PLLA and PLLA with 2 wt % ZnO scaffolds (denoted as PLLA/ZnO) were also prepared using the same procedure for comparison purposes. Among them, a PLLA/ZnO scaffold was selected to compare the ability of the PLLA/C–ZnO scaffold for the slow and controlled release of Zn ions.

### 2.4. Analysis and Characterization

The sample morphologies were captured via scanning electron microscopy (SEM, Zeiss EVO-18, Cambridge, England). Briefly, a few powder samples were ultrasonically dispersed in ethanol; the mixture was then dripped onto the sample holder using conductive tape and stored for drying. After that, the samples were sprayed with gold for 30 s and then fixed to the SEM sample holder. The C–ZnO powder could be directly observed without the gold coating. Finally, the samples were analyzed using SEM.

The morphologies and elemental distributions of the samples were examined via transmission electron microscopy (TEM, JEOL-2100FS, Tokyo, Japan) with energy-dispersive X-ray spectroscopy (EDS). Briefly, the powder samples were ultrasonically dispersed in ethanol (~0.02 mg/mL) and then dropped onto the copper grid (~5 μL). The supernatant liquid was extracted, utilizing a piece of filter paper. After drying, the samples were loaded onto the TEM sample holder for observations. The phase compositions were investigated using X-ray diffraction (XRD, D8 advance, Karlsruhe, Germany ) diffractometer. The chemical compositions were investigated using X-ray photoelectron spectroscopy (XPS) with an EscaLab Xi+ instrument.

### 2.5. Biodegradability and Zn Ion Release Behavior

The biodegradability of the bone scaffold is crucial for the simultaneous growth of defective bone tissue and scaffold degradation. In this study, the biodegradability of the scaffolds was determined by immersing the scaffold samples in phosphate buffer solution (PBS) for designated periods, and the weight loss was monitored. Specifically, the scaffold samples (φ13 × 3 mm) were incubated in 10 mL of PBS solution at 37 °C in an incubator. After 1, 2, 3, and 4 weeks of incubation, the scaffold samples were extracted, followed by washing with PBS and drying at 50 °C. Subsequently, the scaffold samples were weighed to calculate the weight loss [39]:

(1) $$\mathrm{Weight}\;\mathrm{loss}=\frac{w_{0}-w_{t}}{w_{0}}\times 100\%$$

where *W*_0_ and *W_t_* represented the initial weight and the weight of the immersed samples up to week *t* (1, 2, 3, and 4 weeks), respectively.

The Zn ion release behavior of the scaffold was evaluated by quantifying Zn ion concentration at 1, 3, 5, 7, 14, 21, and 28 days, using an inductively coupled plasma optical emission spectrometer (ICP-OES, Spectro Blue Sop, Spectro, Germany). Initially, the scaffold samples were cultured in 10 mL of deionized water. After each time interval, the solution used to immerse the scaffold was collected to determine the Zn ion concentration by ICP-OES. Furthermore, the cumulative amount of Zn ion release within a 28-day period from the scaffold was calculated by accumulating the non-cumulative release of the corresponding days.

### 2.6. Cell Culture and Bioactivity Characterizations

Prior to conducting cell tests, the scaffold samples (φ13 × 3 mm) were immersed in 70% ethanol for 0.5 h, washed with PBS solution 3 times, and sterilized with ultraviolet light. After that, the scaffold samples were cultured in a cell culture medium for 30 min. In this study, human osteoblast-like MG-63 cells were utilized as test cells, which were proliferated in Dulbecco’s Modified Eagle Medium (DMEM) supplemented with 10% FBS and 1% penicillin/streptomycin. After that, the cells were passaged and collected.

Cell adhesion morphologies: initially, the harvested cells (10^5^ cells/mL) were seeded on the scaffold samples for 4 and 7 days. After the completion of co-culture, the scaffold samples were extracted from the medium, refreshed 3 times, and preserved by 2.5% glutaraldehyde. The scaffold samples were then dehydrated with ethanol. The scaffold samples were observed using SEM to obtain the cell adhesion morphologies.

Staining of live/dead cells: typically, the harvested cells (10^5^ cells/mL) were seeded onto the scaffolds. After 4 and 7 days of incubation, the cells were stained with calcein AM and propidium iodide (PI), which dyed the live cells green and the dead cells red, respectively. The stained cells were observed using fluorescence microscopy (Olympus Corporation, Tokyo, Japan).

CCK-8 assay: 10^5^ cells/mL of MG-63 cells were co-cultured on the scaffold samples. On days 4 and 7, 10 µL of CCK-8 reagent was co-cultured with the scaffolds for 4 h. After that, the optical density (OD, 450 nm) value of each sample was determined using a microplate reader.

Alizarin Red staining: The mineralization nodules of MG-63 cells were evaluated by Alizarin Red staining to verify the osteogenic ability of the scaffold. Initially, 10^5^ cells/mL of MG-63 cells were seeded on the scaffolds. After 7 days of co-culture, the scaffolds were extracted and fixed with 4% paraformaldehyde, then rinsed with PBS. Afterward, the scaffolds were stained with 0.04 M of Alizarin Red for 10 min. Finally, the mineralization nodules of MG-63 cells on the scaffolds were examined under a light microscope to obtain staining images.

### 2.7. Antibacterial Ability

*Escherichia coli* (*E. coli*, ATCC 6538) was selected as a test bacterium to identify the antibacterial ability of the scaffolds. Each scaffold sample was divided into dark and light groups for comparison (with or without light as the control variable). Briefly, *E. coli* with a density of 10^6^ CFU/mL was cultivated with the scaffolds in a centrifuge tube for 1 day. Specifically, the samples in the light group were treated with Xe light for 20 min. Afterward, the scaffolds were extracted from the centrifuge tube, washed gently with PBS, and shaken with l mL of lysogeny broth medium for 10 min. Then, the bacterial suspension was diluted 10^4^-fold. Subsequently, 100 μL of the diluted solution was evenly placed on an agar plate. After 24 h of incubation at 37 °C, photographs of the plates were captured using a digital camera. In order to further quantify the antibacterial properties of the scaffolds, the OD value of the diluted bacterial solution was measured with a microplate reader (Beckman, USA). The antibacterial rate was also computed [40]:

(2) $$\mathrm{Antibacterial}\;\mathrm{rate}=\frac{B_{c}-B_{t}}{B_{c}}\times 100\%$$

where *B_c_* and *B_t_* represent the OD values of the negative control and test group, respectively.

#### Statistical Analysis

Student’s *t*-test for independent samples was utilized to examine the statistical differences. The data in the experiments were indicated with (*) for *p* < 0.05 and (**) for *p* < 0.001.

## 3. Results and Discussion

### 3.1. Morphology and Structure of C–ZnO

**Figure 2:**
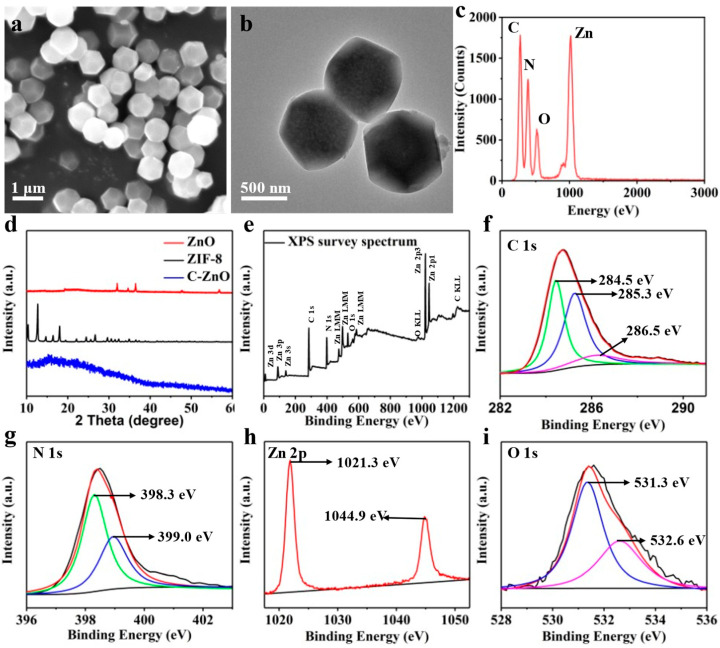
(**a**) SEM image, (**b**) TEM image, and (**c**) corresponding EDS spectrum. (**d**) XRD patterns. (**e**) XPS spectrum and corresponding high-resolution XPS spectra of (**f**) C 1s, (**g**) N 1s, (**h**) Zn 2p, and (**i**) O 1s.

The morphologies of the as-obtained samples were examined via SEM and TEM (Figure 1a–c). Clearly, ZIF-8 exhibited a characteristic rhombic dodecahedron morphology, with a particle size of about 800 nm (Figure 2a), which was comparable with previously published results [41]. After calcination, C–ZnO retained a similar morphology to ZIF-8 (Figure 2b), despite having smaller particles than ZIF-8 (ca. 600 nm). The corresponding EDS results of the C–ZnO are also shown in Figure 2c. The results indicated that C–ZnO was made up of four elements: C, N, O, and Zn.

The XRD results are shown in Figure 2d to characterize the phase structure of the samples. It can clearly be seen that the XRD patterns of ZnO and ZIF-8 were consistent with the standard cards [42], indicating a successful synthesis of the samples. After the carbonization of ZIF-8, the disappearance of the ZIF-8 peaks in the C–ZnO samples suggest a destroyed crystal structure of ZIF-8. In addition, there was also no prominent peak of ZnO in the C–ZnO samples, implying the presence of an amorphous structure of ZnO. This was because the grain growth of ZnO was confined by carbon during carbonization, thereby retarding the crystallization of ZnO [43]. Although no apparent peaks of ZnO can be observed, the Zn element is converted to ZnO at 800 °C, according to previous reports [44]. Subsequent XPS results further confirmed the existence of ZnO by the binding energy position of the Zn–O bond.

The XPS patterns depicted in Figure 2e demonstrate the presence of C, N, O, and Zn peaks. The C 1s spectra in Figure 2f were divided into peaks at 284.5, 285.3, and 286.5 eV, which correspond to C sp^3^–C sp^3^, C sp^2^–C sp^2^, and C=N, respectively [45]. It is noteworthy that the existence of C=N bonds revealed that the N was doped in carbon [46]. Moreover, the high-resolution XPS N 1s spectra in Figure 2g are divided into 398.3 eV (pyridinic N) and 399.0 eV (Zn–N) [47]. The Zn 2p spectra in Figure 2h were divided into peaks at 1021.3 and 1044.9 eV, which can be attributed to Zn 2p3/2 and Zn 2p1/2, respectively [48]. The gap (23.1 eV) between the two peaks can be used as evidence that Zn^2+^ ions were present in the C–ZnO [49]. The O 1s spectra (Figure 2i) had two peaks at 531.3eV (O^2−^ ions of ZnO) and 532.6 eV (oxygen vacancy) [50]. Consequently, the Zn–N and C=N linkages indicate that ZnO was anchored onto the C skeleton by a Zn–N linkage, which maintained the stability of the structure and inhibited the rapid release of ZnO from the scaffold. According to the above results, carbon–ZnO (C–ZnO) nanoparticles were successfully constructed, in which ZnO was embedded in the carbon matrix via Zn–N linkage.

### 3.2. Biodegradability and Zn Ion Release Behavior

**Figure 3:**
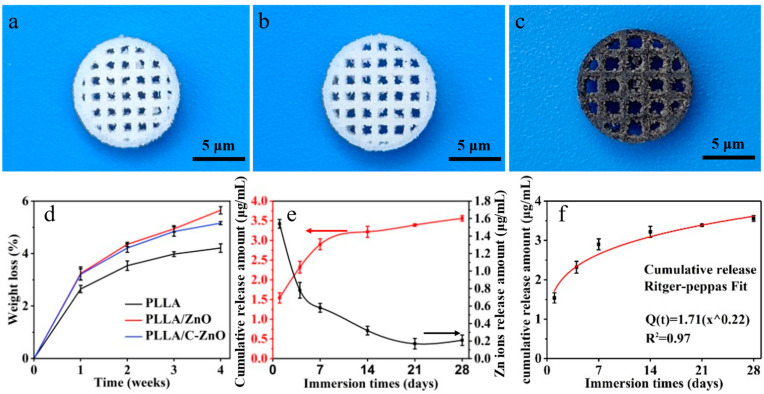
The real images of the (**a**) PLLA, (**b**) PLLA/ZnO, and (**c**) PLLA/C-ZnO scaffolds. (**d**) Weight loss of the scaffolds. (**e**) Cumulative and non-cumulative release amounts of Zn ions. (**f**) Ritger–Peppas fitting of the release curve of the PLLA/C-ZnO scaffold.

The real images of thePLLA, PLLA/ZnO, and PLLA/C-ZnO scaffolds were exhibited in Figure 3a–c. All the scaffold exhibited the interconnected porous shape with pore sizes of approximately 1 μm, which was benefited for nutrient exchange as well as cell adhesion, proliferation, and differentiation. Furthermore, the PLLA and PLLA/ZnO scaffold were white, while the PLLA/C-ZnO scaffold was black due to the addition of black C/ZnO. The degradation behavior was investigated by measuring the weight loss of the scaffolds immersed in PBS for a predetermined time (1, 2, 3, and 4 weeks). As shown in Figure 3d, all the scaffolds kept losing weight with an increase in immersion time. After 4 weeks of immersion, the PLLA scaffold lost 4.21% of its weight, while the PLLA/ZnO and PLLA/C–ZnO scaffolds lost 5.65 and 5.16% of their weight, respectively. The results indicate that ZnO and C–ZnO slightly accelerated the degradation rate of the PLLA matrix. This was likely to be because hydrophilic ZnO made it easier for water molecules to make contact with the scaffolds, thus accelerating the erosion of water molecules on the molecular chain of PLLA. As a result, the degradation rate of the scaffold was improved [51]. The results indicate that PLLA/C-ZnO scaffolds have less weight loss than PLLA/ZnO, but the difference is marginal. In fact, although the proportions of ZnO and C-ZnO in the scaffold were the same (2 wt % in the scaffold), the absolute amount of ZnO in the PLLA/ZnO scaffold was slightly higher than that in the PLLA/C-ZnO scaffold due to the presence of C. ZnO is intrinsically more hydrophilic, which makes it easier for water molecules to attack the scaffold, thus accelerating degradation.

The release behavior of the Zn ion from the PLLA/C–ZnO scaffold was also studied using an immersion test. Specifically, the cumulative release curve and non-cumulative release curve are represented by red and black lines, respectively. As shown in Figure 3e, clearly, the PLLA/C–ZnO scaffold exhibited a relatively high Zn ion release of 1.54 μg/mL on the first day. After 1 week, the release rates slowed down moderately and tended to be stable. Encouragingly, 0.21 μg/mL of the Zn ions was still released from the PLLA/C–ZnO scaffold after 4 weeks, which was close to the release rate at 1 week of 0.58 μg/mL. Additionally, the cumulative release of Zn ions kept a steady increase after 4 weeks. Hence, the results demonstrated that the scaffold could slow and regulate the release of Zn ions. It was reported that Zn ion concentration in the range of 0.18 μg/mL to 1.8 μg/mL has a positive effect on osteogenesis [52,53]. Therefore, the release of Zn ions from the PLLA/C–ZnO scaffold was within a suitable range for use in the body.

Ritger–Peppas fitting was used to further understand the ion release behavior of the PLLA/C–ZnO scaffold [54]:

(3) $$\frac{M_{t}}{M_{\infty }}{=kt}^{n}$$

where *M_t_* and *M_∞_* are the cumulative release of Zn ions at the time (*t*) and the full release, respectively; *k* and *n* characterize the release constant and mechanism, respectively. The release mechanism depends on the value of *n*: (1) *n* < 0.45 (Fickian diffusion); (2) 0.45 < *n* < 0.89 (non-Fickian diffusion); (3) *n* > 0.89 (skeletal dissolution) [55]. After fitting, the obtained correlation coefficient (R^2^) was 0.97, indicating a satisfactory fitting. In general, the closer the value of R^2^ is to 1, the better the fitting effect is. The fitting results are shown in Figure 3f. The obtained *n* was 0.22, assigned to Fickian diffusion, indicating that the PLLA/C–ZnO scaffold could sustain the release of Zn ions.

Therefore, the PLLA/C–ZnO scaffold exhibited the sustained release of Zn ions with a reasonable concentration, which implies that it can be expected to play a role in promoting bone formation without sacrificing the biocompatibility of the scaffold. On the one hand, the ability of the PLLA/C–ZnO scaffold to achieve sustained release of the Zn ion could be explained by the fact that the Zn-N bonding between ZnO and the carbon matrix could impede the molecular motion of ZnO, thereby maintaining the structural stability of ZnO and achieving the slow release of Zn ions. On the other hand, a PLLA scaffold also could as a barrier to delay ZnO contact with the surrounding environment, which further ensures the continual release of Zn ions.

### 3.3. Cellular Behaviors

**Figure 4:**
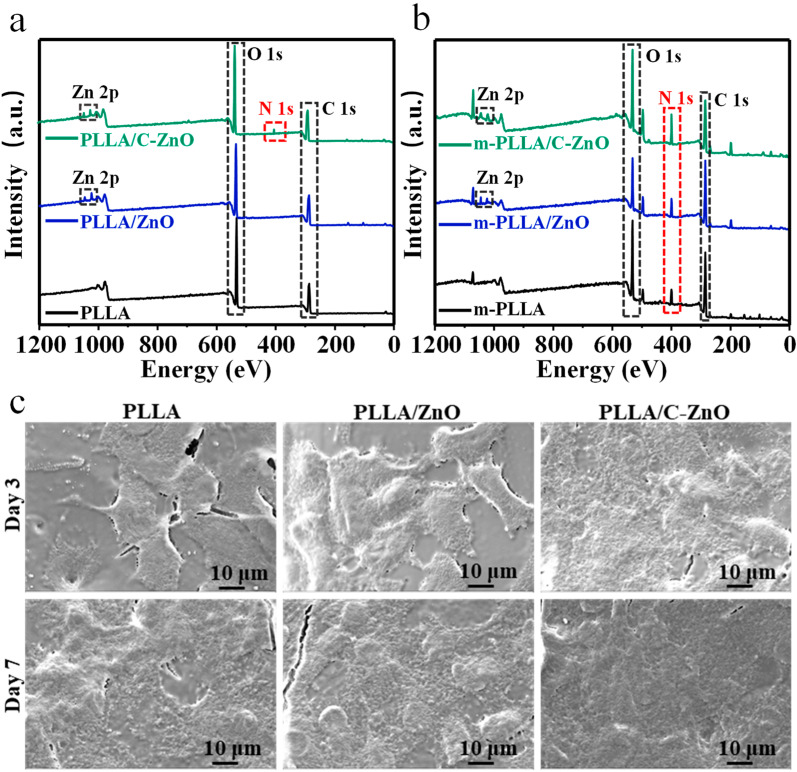
XPS spectra for (**a**) the initial scaffolds and (**b**) the scaffolds after being immersed in the cell media. (**c**) Cell adhesion morphologies on the scaffolds.

To investigate the adsorption capacity of the scaffolds in terms of nitrogen-containing nutrients, XPS tests were conducted to determine the elemental composition of the scaffolds. In particular, the immersed scaffolds were renamed m-PLLA, m-PLLA/ZnO, and m-PLLA/C–ZnO for comparison. The XPS results are shown in Figure 4a,b. After immersion, the nitrogen contents in all scaffolds increased considerably compared to the initial nitrogen contents of the scaffolds. This is because the scaffolds have the ability to absorb nitrogen-containing nutrients from media, such as proteins, peptides, or amino acids [56], thus increasing their nitrogen content. In comparison with the m-PLLA scaffold, the m-PLLA/ZnO scaffold displayed marginal changes in nitrogen content. However, higher nitrogen content was detected on the m-PLLA/C–ZnO scaffold. The results revealed that carbons were beneficial for the scaffolds’ absorption of these nutrient molecules, which is advantageous for the growth and differentiation of cells.

The cell adhesion morphologies are shown in Figure 4c. After culturing for 3 days, it could be seen that the cells were firmly adhered to the scaffold and displayed flat and stretched shapes. After culturing for 7 days, the number of cells on both scaffolds increased. In addition, the number and thickness of cells on the PLLA/C–ZnO scaffold rose dramatically, indicating that the PLLA/C–ZnO scaffold offers excellent cytocompatibility.

**Figure 5:**
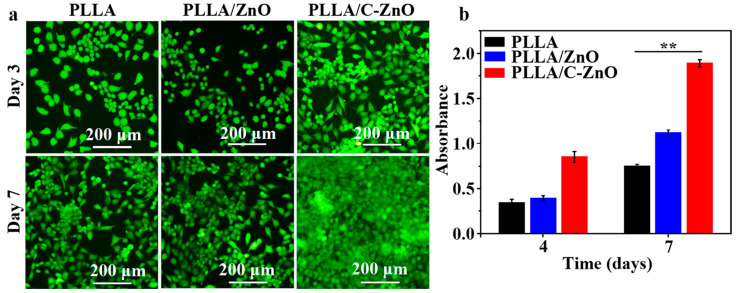
The fluorescence assay results: (**a**) fluorescence images and (**b**) the results of the CCK-8 assay (*n* = 2, ** *p* < 0.01).

The cell toxicity of the scaffolds was assessed using fluorescence assays. The results are shown in Figure 5a. On day 3, the PLLA and PLLA/ZnO scaffolds exhibited only a small number of cells. Comparatively, the PLLA/C–ZnO scaffold groups displayed a comparatively high number of living cells with the PLLA scaffold. As the culture period progressed, the number of cells in all scaffolds increased. Among them, the PLLA/C–ZnO scaffold displayed the largest number of living cells over time, suggesting that the PLLA/C–ZnO scaffold offered superior biocompatibility.

The CCK-8 results are shown in Figure 5b. It can be observed that the PLLA and PLLA/ZnO scaffolds displayed a minor rise in absorbance (OD value) as the culture time progressed. However, the PLLA/C–ZnO scaffold demonstrated a considerable increase in OD value. Moreover, the OD value of the PLLA/C–ZnO scaffold was significantly increased compared with other scaffolds during the same culture period, indicating that the PLLA/C–ZnO scaffold offered a better environment for proliferation.

**Figure 6:**
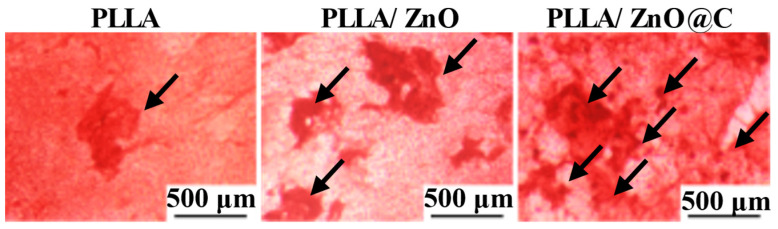
Images taken after the Alizarin Red staining procedure, the red precipitates were pointed by the black arrows.

The results of the Alizarin Red staining are shown in Figure 6. Clearly, the red precipitates (indicated by black arrows) are visible in all scaffolds. It was noteworthy that mineral deposition was dramatically increased in the PLLA/C–ZnO scaffold compared to other groups, mostly as a result of controlled Zn ion release and the improvement of the adsorption capacity of the PLLA/C–ZnO scaffold for nutrients. The aforementioned findings revealed that the incorporation of C–ZnO significantly increased the cell activity and mineralization of the PLLA scaffold, which indicates that the PLLA/C–ZnO scaffold is a potential bone substitute.

**Figure 7:**
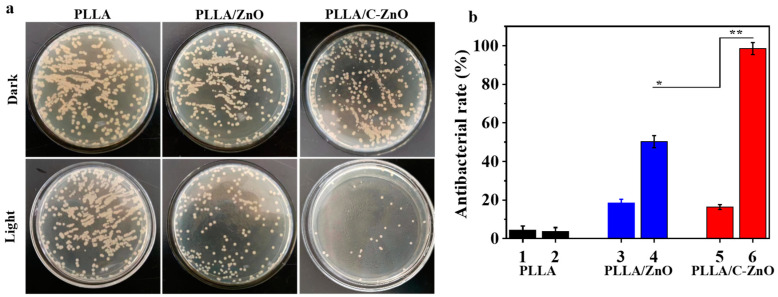
(**a**) The images of bacterial colonies in the dark and light groups. (**b**) Antibacterial rates, where 1, 3, 5 belong to the dark group, and 2, 4, and 6 belong to the light group (*n* = 3, * *p* < 0.05, ** *p* < 0.01).

Bacterial infection often occurs during bone transplantation, which is one of the main reasons for the failure of bone transplants. Therefore, it is necessary to study the antibacterial function of the scaffold to prevent bacterial infection. As a potential new antibacterial method, photodynamic antibacterial effects have demonstrable advantages in non-drug resistance and non-metal ion poisoning. The photocatalytic antibacterial effect of the PLLA/C–ZnO scaffold was investigated using the bacterial spread plate method. The bacterial colonies were photographed (see Figure 7a), and the antibacterial rate is shown in Figure 7b. A substantial number of bacterial colonies were apparently discovered in the dark group, demonstrating that all the scaffolds lacked antibacterial activity in a dark environment. In the light group, there were still numerous bacterial colonies on the PLLA and PLLA/ZnO scaffolds, demonstrating that the scaffolds still had no obvious antibacterial effects under Xe light. Conversely, the bacterial colonies were significantly reduced in the case of the PLLA/C–ZnO scaffold under Xe light, indicating powerful antibacterial performance. This efficient antibacterial effect was because the photo-generated electrons of ZnO were transferred to carbon, thus preventing the recombination of electrons and holes. The increased electrons and holes would further react with water and oxygen, which, in turn, improved the efficiency of ROS generation, which could damage the bacterial membrane and cause bacterial death. The corresponding antibacterial rate also confirmed the conclusion. Under light irradiation, the PLLA/C–ZnO exhibited an antibacterial rate of over 95%, while other scaffolds had an insufficient antibacterial rate.

## 4. Conclusions

To endow a PLLA scaffold with favorable bioactivity, MOF-derived carbon–ZnO was successfully produced by carbonizing ZIF-8. Then, C–ZnO was introduced into the PLLA scaffolds using an SLS 3D printing technique. The Zn ion release kinetics demonstrated that the scaffolds were capable of continuously releasing Zn ions with a concentration above 0.21 μg/mL for more than 28 days, which successfully stimulated cell adhesion and proliferation and boosted the osteogenic differentiation capacity. Moreover, C–ZnO could endow the scaffold with photodynamic antibacterial capacity (95% of the antibacterial rate). Therefore, the PLLA/C–ZnO scaffold offers favorable bioactivity and antibacterial properties, which is expected to make them a viable candidate for bone repair.

## Data Availability

Data not available/Data will be made available on request.

## References

[1] Zhou G., Wang F., Lin G., Tang B., Li X., Ding X., Wang W., Zhang J., Shi Y. (2023). Novel coatings for the continuous repair of human bone defects. Colloids Surf. B Biointerfaces.

[2] Alonzo M., Primo F.A., Kumar S.A., Mudloff J.A., Dominguez E., Fregoso G., Ortiz N., Weiss W.M., Joddar B. (2021). Bone tissue engineering techniques, advances, and scaffolds for treatment of bone defects. Curr. Opin. Biomed. Eng..

[3] Bose S., Sarkar N., Banerjee D. (2021). Natural medicine delivery from biomedical devices to treat bone disorders: A review. Acta Biomater..

[4] Ho-Shui-Ling A., Bolander J., Rustom L.E., Johnson A.W., Luyten F.P., Picart C. (2018). Bone regeneration strategies: Engineered scaffolds, bioactive molecules and stem cells current stage and future perspectives. Biomaterials.

[5] Patel B.B., Sharifi F., Stroud D.P., Montazami R., Hashemi N.N., Sakaguchi D.S. (2019). 3D microfibrous scaffolds selectively promotes proliferation and glial differentiation of adult neural stem cells: A platform to tune cellular behavior in neural tissue engineering. Macromol. Biosci..

[6] Seidi A., Ramalingam M., Elloumi-Hannachi I., Ostrovidov S., Khademhosseini A. (2011). Gradient biomaterials for soft-to-hard interface tissue engineering. Acta Biomater..

[7] Woo K.M., Chen V.J., Ma P.X. (2003). Nano-fibrous scaffolding architecture selectively enhances protein adsorption contributing to cell attachment. J. Biomed. Mater. Res..

[8] Dubinenko G., Zinoviev A., Bolbasov E., Kozelskaya A., Shesterikov E., Novikov V., Tverdokhlebov S. (2021). Highly filled poly (l-lactic acid)/hydroxyapatite composite for 3D printing of personalized bone tissue engineering scaffolds. J. Appl. Polym. Sci..

[9] Meng J., Boschetto F., Yagi S., Marin E., Adachi T., Chen X., Pezzotti G., Sakurai S., Yamane H., Xu H. (2021). Design and manufacturing of 3D high-precision micro-fibrous poly (l-lactic acid) scaffold using melt electrowriting technique for bone tissue engineering. Mater. Des..

[10] Dinarvand P., Seyedjafari E., Shafiee A., Jandaghi A.B., Doostmohammadi A., Fathi M.H., Farhadian S., Soleimani M. (2011). New Approach to Bone Tissue Engineering: Simultaneous Application of Hydroxyapatite and Bioactive Glass Coated on a Poly(l-lactic acid) Scaffold. ACS Appl. Mater. Interfaces.

[11] Liu S., Qin S., He M., Zhou D., Qin Q., Wang H. (2020). Current applications of poly(lactic acid) composites in tissue engineering and drug delivery. Compos. Part B Eng..

[12] Tyler B., Gullotti D., Mangraviti A., Utsuki T., Brem H. (2016). Polylactic acid (PLA) controlled delivery carriers for biomedical applications. Adv. Drug Deliv. Rev..

[13] O’Connor J.P., Kanjilal D., Teitelbaum M., Lin S.S., Cottrell J.A. (2020). Zinc as a Therapeutic Agent in Bone Regeneration. Materials.

[14] Hoppe A., Güldal N.S., Boccaccini A.R. (2011). A review of the biological response to ionic dissolution products from bioactive glasses and glass-ceramics. Biomaterials.

[15] Shi Z.-Z., Gao X.-X., Zhang H.-J., Liu X.-F., Li H.-Y., Zhou C., Yin Y.-X., Wang L.-N. (2020). Design biodegradable Zn alloys: Second phases and their significant influences on alloy properties. Bioact. Mater..

[16] Kabir H., Munir K., Wen C., Li Y. (2020). Recent research and progress of biodegradable zinc alloys and composites for biomedical applications: Biomechanical and biocorrosion perspectives. Bioact. Mater..

[17] Qin Y., Liu A., Guo H., Shen Y., Wen P., Lin H., Xia D., Voshage M., Tian Y., Zheng Y. (2022). Additive manufacturing of Zn-Mg alloy porous scaffolds with enhanced osseointegration: In vitro and in vivo studies. Acta Biomater..

[18] Zhong Z., Wu X., Wang Y., Li M., Li Y., Liu X., Zhang X., Lan Z., Wang J., Du Y. (2022). Zn/Sr dual ions-collagen co-assembly hydroxyapatite enhances bone regeneration through procedural osteo-immunomodulation and osteogenesis. Bioact. Mater..

[19] Hernández-Escobar D., Champagne S., Yilmazer H., Dikici B., Boehlert C.J., Hermawan H. (2019). Current status and perspectives of zinc-based absorbable alloys for biomedical applications. Acta Biomater..

[20] Chopra D., Gulati K., Ivanovski S. (2021). Understanding and optimizing the antibacterial functions of anodized nano-engineered titanium implants. Acta Biomater..

[21] Bowen P.K., Shearier E.R., Zhao S., Guillory R.J., Zhao F., Goldman J., Drelich J.W. (2016). Biodegradable Metals for Cardiovascular Stents: From Clinical Concerns to Recent Zn-Alloys. Adv. Healthc. Mater..

[22] Wiesmann N., Tremel W., Brieger J. (2020). Zinc oxide nanoparticles for therapeutic purposes in cancer medicine. J. Mater. Chem. B.

[23] Jiang S., Lin K., Cai M. (2020). ZnO Nanomaterials: Current Advancements in Antibacterial Mechanisms and Applications. Front. Chem..

[24] Jiang Y., Xiong Z., Huang J., Yan F., Yao G., Lai B. (2022). Effective *E. coli* inactivation of core-shell ZnO@ ZIF-8 photocatalysis under visible light synergize with peroxymonosulfate: Efficiency and mechanism. Chin. Chem. Letters.

[25] Mahamuni-Badiger P.P., Patil P.M., Badiger M.V., Patel P.R., Gadgil B.S.T., Pandit A., Bohara R.A. (2020). Biofilm formation to inhibition: Role of zinc oxide-based nanoparticles. Mater. Sci. Eng. C.

[26] Sanati S., Abazari R., Morsali A., Kirillov A.M., Junk P.C., Wang J. (2019). An Asymmetric Supercapacitor Based on a Non-Calcined 3D Pillared Cobalt(II) Metal–Organic Framework with Long Cyclic Stability. Inorg. Chem..

[27] Bai S., Liu C., Luo R., Chen A. (2018). Metal organic frameworks-derived sensing material of SnO_2_/NiO composites for detection of triethylamine. Appl. Surf. Sci..

[28] Chen B., Yang X., Zeng X., Huang Z., Xiao J., Wang J., Zhan G. (2020). Multicomponent metal oxides derived from Mn-BTC anchoring with metal acetylacetonate complexes as excellent catalysts for VOCs and CO oxidation. Chem. Eng. J..

[29] Guo S., Zhao Y., Yuan H., Wang C., Jiang H., Cheng G.J. (2020). Ultrafast Laser Manufacture of Stable, Efficient Ultrafine Noble Metal Catalysts Mediated with MOF Derived High Density Defective Metal Oxides. Small.

[30] Liu Y., Cheng H., Cheng M., Liu Z., Huang D., Zhang G., Shao B., Liang Q., Luo S., Wu T. (2021). The application of Zeolitic imidazolate frameworks (ZIFs) and their derivatives based materials for photocatalytic hydrogen evolution and pollutants treatment. Chem. Eng. J..

[31] Salunkhe R.R., Kaneti Y.V., Yamauchi Y. (2017). Metal–Organic Framework-Derived Nanoporous Metal Oxides toward Supercapacitor Applications: Progress and Prospects. ACS Nano.

[32] Li Y., Zhang J., Chen Q., Xia X., Chen M. (2021). Emerging of Heterostructure Materials in Energy Storage: A Review. Adv. Mater..

[33] Kang W., Wang Y., Wan Y., Tuo Y., Wang X., Sun D. (2021). Embedding anion-doped Fe7S8 in N-doped carbon matrix and shell for fast and stable sodium storage. Mater. Chem. Phys..

[34] Xia Y., Fan X., Yang H., Li L., He C., Cheng C., Haag R. (2020). ZnO/Nanocarbons-Modified Fibrous Scaffolds for Stem Cell-Based Osteogenic Differentiation. Small.

[35] Johnston H.J., Hutchison G.R., Christensen F.M., Peters S., Hankin S., Aschberger K., Stone V. (2010). A critical review of the biological mechanisms underlying the in vivo and in vitro toxicity of carbon nanotubes: The contribution of physicochemical characteristics. Nanotoxicology.

[36] Qi K., Cheng B., Yu J., Ho W. (2017). Review on the improvement of the photocatalytic and antibacterial activities of ZnO. J. Alloys Compd..

[37] Ravichandiran P., Masłyk M., Sheet S., Janeczko M., Premnath D., Kim A.R., Park B.H., Han M.K., Yoo D.J. (2019). Synthesis and antimicrobial evaluation of 1, 4-naphthoquinone derivatives as potential antibacterial agents. ChemistryOpen.

[38] Subramaniyan S.A., Sheet S., Vinothkannan M., Yoo D.J., Lee Y.S., Belal S.A., Shim K.S. (2018). One-pot facile synthesis of Pt nanoparticles using cultural filtrate of microgravity simulated grown *P. chrysogenum* and their activity on bacteria and cancer cells. J. Nanosci. Nanotechnol..

[39] Shuai C., Yang W., Feng P., Peng S., Pan H. (2021). Accelerated degradation of HAP/PLLA bone scaffold by PGA blending facilitates bioactivity and osteoconductivity. Bioact. Mater..

[40] Ji H., Zhao M.-C., Xie B., Zhao Y.-C., Yin D., Gao C., Shuai C., Atrens A. (2021). Corrosion and antibacterial performance of novel selective-laser-melted (SLMed) Ti-xCu biomedical alloys. J. Alloys Compd..

[41] Yang R., Yan X., Li Y., Zhang X., Chen J. (2017). Nitrogen-doped porous carbon-ZnO nanopolyhedra derived from ZIF-8: New materials for photoelectrochemical biosensors. ACS Appl. Mater. Interfaces.

[42] Lv R., Zhang Q., Wang W., Lin Y., Zhang S. (2021). ZnO@ ZIF-8 Core-Shell Structure Gas Sensors with Excellent Selectivity to H_2_. Sensors.

[43] Duarah R., Karak N. (2019). Hyperbranched polyurethane/reduced carbon dot-zinc oxide nanocomposite-mediated solar-assisted photocatalytic degradation of organic contaminant: An approach towards environmental remediation. Chem. Eng. J..

[44] Samuel E., Joshi B., Kim M.-W., Kim Y.-I., Swihart M.T., Yoon S.S. (2019). Hierarchical zeolitic imidazolate framework-derived manganese-doped zinc oxide decorated carbon nanofiber electrodes for high performance flexible supercapacitors. Chem. Eng. J..

[45] Wang A., Ni J., Wang W., Wang X., Liu D., Zhu Q. (2022). MOF-derived N-doped ZnO carbon skeleton@ hierarchical Bi_2_MoO_6_ S-scheme heterojunction for photodegradation of SMX: Mechanism, pathways and DFT calculation. J. Hazard. Mater..

[46] Tabassum H., Qu C., Cai K., Aftab W., Liang Z., Qiu T., Mahmood A., Meng W., Zou R. (2018). Large-scale fabrication of BCN nanotube architecture entangled on a three-dimensional carbon skeleton for energy storage. J. Mater. Chem. A.

[47] Fan C., Tang Y., Wang H., Huang Y., Xu F., Yang Y., Huang Y., Rong W., Lin Y. (2022). ZIF-90 with biomimetic Zn–N coordination structures as an effective nanozyme to mimic natural hydrolase. Nanoscale.

[48] Wahab R., Hwang I., Kim Y.-S., Shin H.-S. (2011). Photocatalytic activity of zinc oxide micro-flowers synthesized via solution method. Chem. Eng. J..

[49] Panigrahy B., Aslam M., Bahadur D. (2010). Aqueous Synthesis of Mn- and Co-Doped ZnO Nanorods. J. Phys. Chem. C.

[50] Liu X., Wang L.-S., Ma Y., Zheng H., Lin L., Zhang Q., Chen Y., Qiu Y., Peng D.-L. (2017). Enhanced microwave absorption properties by tuning cation deficiency of perovskite oxides of two-dimensional LaFeO3/C composite in X-band. ACS Appl. Mater. Interfaces.

[51] Abdalkarim S.Y.H., Yu H.-Y., Song M.-L., Zhou Y., Yao J., Ni Q.-Q. (2017). In vitro degradation and possible hydrolytic mechanism of PHBV nanocomposites by incorporating cellulose nanocrystal-ZnO nanohybrids. Carbohydr. Polym..

[52] Wang R., He X., Gao Y., Zhang X., Yao X., Tang B. (2017). Antimicrobial property, cytocompatibility and corrosion resistance of Zn-doped ZrO_2_/TiO_2_ coatings on Ti6Al4V implants. Mater. Sci. Eng. C.

[53] Qian G., Zhang L., Wang G., Zhao Z., Peng S., Shuai C. (2021). 3D Printed Zn-doped Mesoporous Silica-incorporated Poly-L-lactic Acid Scaffolds for Bone Repair. Int. J. Bioprint..

[54] Lu X., Sun Y., Han M., Chen D., Wang A., Sun K. (2022). Silk fibroin double-layer microneedles for the encapsulation and controlled release of triptorelin. Int. J. Pharm..

[55] Ye S., Jiang L., Su C., Zhu Z., Wen Y., Shao W. (2019). Development of gelatin/bacterial cellulose composite sponges as potential natural wound dressings. Int. J. Biol. Macromol..

[56] Thein-Han W., Saikhun J., Pholpramoo C., Misra R., Kitiyanant Y. (2009). Chitosan–gelatin scaffolds for tissue engineering: Physico-chemical properties and biological response of buffalo embryonic stem cells and transfectant of GFP–buffalo embryonic stem cells. Acta Biomater..

